# Notes and News

**Published:** 1905-09-23

**Authors:** 


### NOTES AND NEWS.

In spite of a warning from their medical officer of health, the inhabitants of Torquay and the neighbourhood have continued to eat shell-fish taken from the rivers, where pollution has been going on for some time. Now an outbreak of typhoid has occurred in the valley of the Teign, and at Torquay a man has died of enteritis and another patient is seriously ill.

The accounts of the Bristol Royal Infirmary Carnival are now published. They reveal the courage which must have animated the promoters. The attendant expenses amounted to upwards of £9,000. Advertising and bill-posting alone cost £985. In the end the Bristol Infirmary gained £15,000, but it must not be forgotten that the carnival scheme received support from Sir George White and Mr. Samuel White, amounting to upwards of £11,500.

A dietetic invention has been recently placed on the market. It is in the form of concentrated meat, which is destined to serve as a conveniently-carried meal. It must not be forgotten, should the facility of consumption tempt the busy man, that the nutritious character of food is not all that is necessary to form a diet. Bulk is a very important factor in digestion. An experiment made some years ago in the American army proved that concentrated food was very unsatisfactory for soldiers on the march.

The Poor-law authorities are evidently placed in a difficult position when a sick pauper certified fit for removal from the infirmary refuses to enter the workhouse. To grant outdoor relief in such circumstances is, as the Local Government Board explained to the Clutton Guardians, tantamount to allowing a pauper choice of in or out-door relief. On the other hand the Guardians incur some responsibility if they withhold aid which is urgently required. In any case the attendance of the parish medical officer would be permitted, and any recommendations made by him could hardly be neglected. We confess we see no direct road out of the difficulty.

The Middlesex Hospital has been re-opened after being closed for eight weeks for repairs.

The International Congress on the Education and Household Management of Infants commenced this week at Liege.

Lord Alverstone has presented a cottage hospital to Shanklin, Isle of Wight, in memory of his son. Princess Henry of Battenberg performed the opening ceremony.

At the present time France has no Poor-law such as we understand it. Poverty has not been recognised as constituting a claim upon the public funds. In July a law was made which will give any needy person over 70 years of age, or one suffering from an incurable infirmity, the right to claim relief. The minimum allowance is 4s. per month, and the maximum 16s. for outdoor relief. Applicants may, in lieu of allowance, be maintained in one of the communal homes or almshouses.

The scheme to provide self-supporting sanatoria for the working classes which the Princess Christian is advocating is an excellent one. There is no doubt that the class which suffers most in times of illness is the lower middle class. The charitable sanatoria and those for the wealthier classes are not open to them. They prefer to, and can as a rule pay a proportion of the cost of treatment, and provided that the organisers of the new scheme secure careful and economic administration, when once the cost of establishing is paid, the institution should certainly be self-supporting.

At the suggestion of the Duchess of Marlborough a bazaar will be held at the Stratford Town Hall on October 26th, 27th, and 28th, 1905, in aid of the Extension Fund of the West Ham and East London Hospital. The committee of the hospital have acquired at considerable cost the High School Buildings adjoining the hospital premises and upwards of £20,000 are required for the equipment of these as an addition to the hospital and the equipment of a nurses' home. The nurses at present sleep in the hospital premises, and it is considered by competent authorities most important that they should have a separate residence. It is extremely urgent that a sum of money should be raised this year in order that the committee may avail itself of the very handsome offer of the Annie Zunz Trustees. These gentlemen have offered that if during the current year the committee raises £7,000 towards the above-mentioned object, they will augment this amount, from funds at their disposal for charitable purposes, by the sum of £3,000, making £10,000 in all. Up to the present only a small portion of the necessary funds has been acquired and the committee are earnestly hoping to make £2,000 or £3,000 by the bazaar. Help in money or in kind should be sent to the hon. secretary, F. J. S. Nicoll, Esq., M.D., 2 Romford Road, Stratford, E., or to the hon. treasurer, George Hay, Esq., J.P., Stratford, E., or to the Secretary at the hospital. The West Ham and East London Hospital is the only general hospital in the midst of an immediate population of 300,000 and in a large and poor district numbering over 500,000, among whom are very few rich people.

